# Intraspecific variation in landform engineering across a restored salt marsh shoreline

**DOI:** 10.1111/eva.13148

**Published:** 2020-11-07

**Authors:** Brittany M. Bernik, Candice Y. Lumibao, Scott Zengel, John Pardue, Michael J. Blum

**Affiliations:** ^1^ Department of Ecology & Evolutionary Biology Tulane University New Orleans LA USA; ^2^ Research Planning, Inc. (RPI) Tallahassee FL USA; ^3^ Department of Civil & Environmental Engineering Louisiana State University Baton Rouge LA USA; ^4^ Department of Ecology & Evolutionary Biology University of Tennessee Knoxville TN USA

**Keywords:** cultivars, *Deepwater Horizon* oil spill, ecosystem genetics, erosion, extended phenotype, functional traits, marsh loss

## Abstract

Ecosystem engineers that modify landforms can be valuable tools for restoring habitat, but their use has frequently resulted in unanticipated outcomes. Departures from expectations might arise because applications discount the possibility that geomorphic processes are influenced by heritable phenotypic variation. We conducted a field‐scale common garden experiment to assess whether shoreline erosion reflects intraspecific variation in the landform engineer *Spartina alterniflora*. Replicated plots on a shoreline denuded by the *Deepwater Horizon* oil spill were revegetated using plants from four genetically distinct sources: the local population, a nonlocal population, and two nursery stocks. We assessed variation in biomass, tissue nutrients, and functional traits alongside soil shear strength, surface elevation, and shoreline erosion rates over 2 years. We found that productivity, traits, nutrient content, and erosion rates varied according to plant provenance. Erosion reflected traits like root architecture more so than coarser metrics of growth. Erosion was significantly higher in plots with nonlocal plants that exhibited lower productivity, likely due to nitrogen limitation. Our results indicate that restoration practices should account for intraspecific variation in landform engineers and that in situ trials should be performed at sites slated for restoration to evaluate donor source suitability, particularly if introductions might modify local populations.

## 1 INTRODUCTION

Ecosystem engineers (i.e., species that create, modify, or maintain habitats; Jones et al., 1994, 1997) can be valuable tools for ecosystem management, especially landform engineers (i.e., species that modify sediment and landform dynamics; Corenblit et al., 2011), which are often deployed to create and restore habitat. Yet use of landform engineers has resulted in a mixed record—applications have frequently yielded conditions that depart from natural precedent or that otherwise result in unexpected consequences (e.g., Strong & Ayres, 2013). It is possible that unanticipated outcomes occur because applications do not adequately account for the influence of a species' ‘extended phenotype’ (sensu Whitham et al., 2003, 2006) on Earth surface processes. Many species exhibit extended phenotypes, which correspond to outcomes of heritable phenotypic variation that transcend individuals. Heritable phenotypic variation in plants can, for instance, influence the composition of biotic communities and ecosystem processes like energy flow and nutrient cycling (Whitham et al., 2003, 2006). Evidence that plant functional traits can influence geomorphology suggests that heritable variation may also mediate Earth surface processes, like the stability and transport of surface materials (Corenblit et al., 2011). If so, then accounting for the possibility that extended phenotypes shape biogeomorphological interactions could improve restoration practices and postrestoration ecosystem management (Bernik, Eppinga, et al., 2018; Bernik, Pardue, et al., 2018).

Unexpected and unintended consequences may arise because restoration practitioners sometimes apply agronomic approaches such as the use of cultivated nursery stocks, hereafter referred to as ‘cultivars’ (e.g., Bernik et al., 2016; Strong & Ayres, 2013). Plants are regularly obtained from nurseries that source or propagate genotypes exhibiting preferred traits (e.g., higher growth rates) or that develop pedigreed lines selected for traits of interest (e.g., that reduce logistical expenses or increase establishment success) (Lesica & Allendorf, 1999; Utomo et al., 2010). Departures from expectations set by natural precedents may emerge because cultivars exhibit a novel or narrower range of trait variation (i.e., compared to more genetically diverse natural populations), or exhibit functional trade‐offs (e.g., greater investment in aboveground growth can reduce belowground investment; Herms & Mattson, 1992) that may only become evident after deployment. Departures also may be amplified if the introduction of cultivars alters the composition of local populations through intraspecific competition or admixture (Strong & Ayres, 2013). Local genets may be outcompeted if, for example, cultivars exhibit attributes that are globally favorable, allowing for more competitive responses to local pressures or stochastic events. Similarly, admixture or hybridization can result in rapid spread and replacement of native flora (e.g., as a result of hybrid vigor, an expanded range of ecological tolerance, or transgressive segregation) followed by whole ecosystem transformation (Bernik et al., 2016; Lesica & Allendorf, 1999; Strong & Ayres, 2013).

Efforts to restore coastal salt marshes highlight the importance of understanding whether heritable phenotypic variation influences landform engineering (Blum et al., 2014). Smooth cordgrass (*Spartina alterniflora*), a well‐recognized landform engineer, is widely used for coastal restoration because it can lend physical integrity to marsh platforms and stabilize shorelines by, for example, increasing soil shear strength by ≥800% (Howes et al., 2010). Belowground growth also can elevate the marsh platform surface (Turner et al., 2002). Aboveground canopy growth can further increase platform elevation by encouraging sediment deposition (Leonard & Luther, 1995; Mudd et al., 2010). Interest in the use of *S. alterniflora* cultivars has been rising, especially since the 2010 *Deepwater Horizon* (DWH) oil spill (Blum et al., 2014; Zengel et al., 2015), in order to support expanding portfolios of restoration projects like those laid out in Louisiana's >$50B Coastal Master Plan (Knott et al., 2012, 2013; Utomo et al., 2010). The prospect of greater cultivar use has been met with some consternation, however, with the many misadventures of introduced *S. alterniflora* serving as cautionary examples of good intentions gone awry (Bernik et al., 2016; Strong & Ayres, 2013).

Determining whether use of *S. alterniflora* (i.e., from a particular source) for restoration might result in undesirable outcomes requires careful evaluation of intraspecific variation in landform engineering. In this study, we undertook a common garden restoration experiment to determine whether physical responses of a marsh shoreline to wind‐wave energy differ according to source material, hereafter referred to as ‘provenance’. We planted replicated plots across a denuded shoreline using locally sourced plants, plants from a nonlocal source, and two cultivar stocks. After 2 years of establishment and growth, we assessed differences in productivity, functional traits as well as soil properties and erosion rates to test the hypothesis that postrestoration landform engineering differs according to plant provenance. We also tested the hypothesis that shorelines stabilized with plants from natural populations withstand erosive forces more so than shorelines planted with cultivars because cultivation results in functional trade‐offs that limit performance.

## 2 MATERIALS AND METHODS

### 2.1 Source materials

We utilized material from four genetically distinct source populations of *S. alterniflora*. We utilized plants from the experimental site in Bay Jimmy (BJ; Plaquemines Parish, LA) to assess the performance of locally sourced *S. alterniflora*. We used plants from a population in Catfish Lake (CL; Lafourche Parish, LA)—which encompasses habitat similar to, but 40 km west of, Bay Jimmy—to assess variation among natural populations within a region. Plant material was gathered from BJ and CL to capture a representative range of genotypic diversity at each source location (Supporting Information). We also planted the Vermilion (V) cultivar, which has been used almost exclusively for marsh restoration across the northern Gulf coast since 1989 because it is a putative clonal monoculture that exhibits consistently high productivity, transplantation survival, and tolerance to inundation and salinity (Fine & Thomassie, 2000; LAPMC, 1989). We additionally used a new outcrossed cultivar (CP), which exhibits comparably high seed production and germination under common garden conditions (Knott et al., 2012, 2013; Utomo et al., 2010). Nurseries at Nicholls State University (NSU) and the Louisiana State University (LSU) AgCenter provided plant material for the V and CP cultivars, respectively. All plant material—consisting of plugs with roots—was brought to the greenhouse, thoroughly rinsed clean, and separated into similarly sized rhizomes with a single stem node for subsequent planting.

We genotyped a representative set of plants using a suite of microsatellite markers (Blum et al., 2004; Sloop et al., 2005) to characterize genetic variation and differentiation according to provenance. Sampling consisted of collecting three green leaf tissue samples (taken from one of the plug locations) from each plot, supplemented with additional 6–16 samples from each source population. DNA was extracted from each sample using a DNEasy plant extraction kit. Samples were scored at 8 microsatellite loci: SPAR3, SPAR5, SPAR7, SPAR8, SPAR14, SPAR16, SPAR18, and SPAR20 (Blum et al., 2004; Sloop et al., 2005). Approximately 10–50 ng of genomic DNA was used as template in 15 µl PCR mixtures that included 1.0 mM MgCl_2_, 166.67 µM each dNTP, 0.5 U hot‐start *Taq* DNA polymerase (MCLAB), 1× PCR buffer (MCLAB), 1 µM each primer, and H_2_O added to attain the final volume. Forward primers were fluorescently labeled with HEX, 6‐FAM, or NED. Amplified products were generated using Eppendorf thermal cyclers (Eppendorf International) programmed to run one cycle at 95°C for 10 min, 35 cycles at 94°C for 45 s, the primer‐specific annealing temperature for 30 s, and 72°C for 90 s, followed by a final extension stage at 72°C for 5 min. Fragment sizes were determined against a GeneScan 600 LIZ standard (Applied Biosystems Inc.) using an ABI 3730xl DNA Analyzer. Electrophoretic output was scored with GeneMarker^®^ software (Soft Genetics LLC). GENALEX v.6.5 was used to identify multilocus matches among genotyped individuals (Peakall & Smouse, 2006) and to calculate plot‐level genotypic richness (G_P_), and the number of different genotypes detected by provenance (G). To examine genetic variation among planted treatments, ARLEQUIN v. 3.5 was used to calculate global and pairwise values of *F*_ST_, and to compute the log‐likelihood of assigning each individual provenance to other treatments (Excoffier & Lischer, 2010). Discriminant analysis of principal components (DAPC) was used to further evaluate the extent of genetic differentiation among the samples according to plot and provenance (Jombart et al., 2010). DAPC was run in R 3.1.1 (R Core Team, 2016) using the ‘adegenet’ package (Jombart, 2008).

### 2.2 Study site and experimental design

**FIGURE 1:**
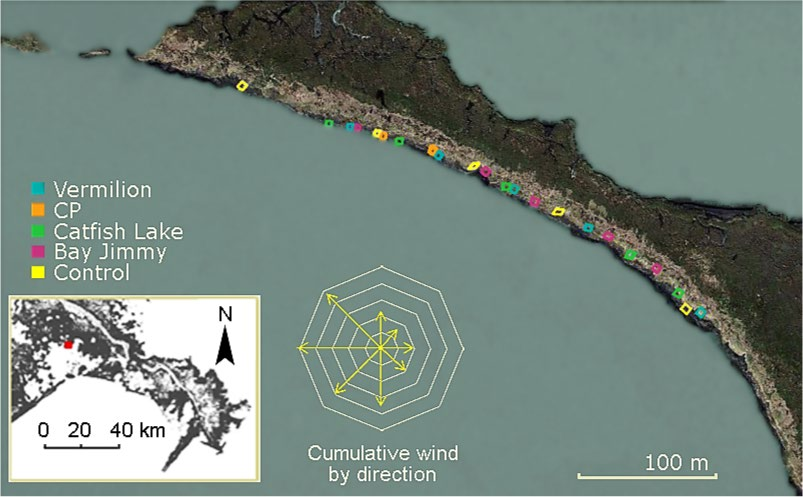
The study site in Bay Jimmy, Louisiana, with reference to cumulative wind gust by direction from September 2012 to September 2013. The colors of outlines on the map distinguish plots planted with the Vermilion cultivar, the CP cultivar, natural stock from the region (Catfish Lake), natural stock from the site (Bay Jimmy), and nonplanted control plots. Map inset depicts the site location relative to the Mississippi River outlet

Following Seliskar et al. (2002), replicate sets of experimental plots were established in July 2011 along 400 m of shoreline in Bay Jimmy (Figure 1), which is located in northern Barataria Bay in southeastern Louisiana (Lat/Long 29°26′37.66″N 89°53′14.74″W). Like fringing marshes elsewhere in northern Barataria Bay, marshes in Bay Jimmy are highly exposed, resulting in rapid rates of peripheral erosion due to wind‐driven wave stress (Bernik, Eppinga, et al., 2018, 2018; Blum et al., 2014; Silliman et al., 2012). Exposure also resulted in wind‐wave delivery of oil during the 2010 *Deepwater Horizon* oil spill (Michel et al., 2013; Nixon et al., 2016; Zengel et al., 2015). By late June 2011, cleanup activities had removed contaminated vegetation and debris from Bay Jimmy, leaving a ~10‐m‐wide devegetated zone across remediated marsh shorelines (Blum et al., 2014; Zengel et al., 2015; Zengel & Michel, 2013).

Using a randomized block design to control for a gradient of wind‐driven wave action along the shoreline (Bernik, Eppinga, et al., 2018), we established five replicate 25‐m^2^ plots with *S. alterniflora* from each source, except for the CP cultivar, which was only planted in two plots due to the scarcity of source material (Figure 1). The plots were established 5 m inland from the waterline (Figure S1) to allow plants time to establish prior to experiencing erosion. Within each plot, 55 bare‐root starter stocks were hand‐planted along measured rows, giving a planting density of 2–3 plants/m^2^. Plants were allowed to establish for a full year prior to the onset of data collection, so that measurements would reflect mature vegetation grown under common garden conditions. By the second year, vegetative cover was continuous for all plots (i.e., approximately 100%) (Figure S1). To assess how restored shoreline (i.e., areas planted with *S. alterniflora*) compared to unrestored shoreline, additional ‘nonplanted’ control plots were kept unvegetated for 1 year, after which natural colonization was allowed to proceed.

### 2.3 Characterization of plant attributes and resource use

We quantified phenotypic variation by provenance following prior studies of heritable trait variation in *S. alterniflora* (Bernik, Pardue, et al., 2018; Seliskar et al., 2002). We assayed a suite of traits known to exhibit heritable variation (Bernik, Pardue, et al., 2018), including traits (e.g., inflorescence length) that are thought to distinguish V and CP cultivars (Fine & Thomassie, 2000; Knott et al., 2012, 2013; Utomo et al., 2010). At the end of the second growing season in November 2013, vegetation in each plot was sampled by harvesting three 10‐cm‐diameter cores that included all aboveground (AG) and belowground (BG) tissue plus soil to a depth of 20 cm. We collected one core from a random location in the interior third, the middle third, and the shoreward third of every plot, respectively (Figure S1). All cores were processed at Tulane University. We harvested AG material to measure shoot height, shoot density, inflorescence count, inflorescence length, and seed weight. We distinguished mature shoots (i.e., with seed heads) from tillers (i.e., <30 cm) and nontillers (i.e., >30 cm). For three mature shoots per core, we recorded shoot diameter, leaf count, and leaf length, standardized by measuring the third leaf down from the seed head. BG material was divided into 10‐cm intervals, after which we separated and cleaned rhizomes, roots, and fine roots. Dry weights were obtained for all AG and BG tissues.

We measured tissue carbon (C) and nitrogen (N) concentrations to examine nutrient accumulation and allocation. Dried AG and BG tissue samples from each core were ground and homogenized with a grinding mill to measure percent mass C and N with a Fisons EA112 Elemental Analyzer (Carlo Erba Instruments; now Thermo Scientific). For comparison, we also analyzed soil C and N concentrations from dried and ground soil samples from both 10‐cm intervals of the BG portion of each core.

### 2.4 Characterization of shoreline erosion, soil shear strength, and surface elevation

For each plot, we estimated erosion rates by measuring the distance between the interior edge of the plot and the respective waterline to the nearest cm along six transects spaced 1 m apart. Measurements were taken in September 2012 and then repeated 3, 6, and 12 months thereafter. Due to the curvature of the shoreline, plots were expected to experience decreasing erosion from west to east according to the equation *P_i,j_ = P_w_* cos *α_j_*, where *P_i_* is the wave power density on impact at plot *j*, *P_w_* is the power density of incoming waves, and *α_j_* is the plot‐specific angle separating the direction of wave propagation from shore normal (Bernik, Eppinga, et al., 2018; Marani et al., 2018, 2011). Thus to standardize across plots, we divided erosion measures by cos *α_j_*. Data from NOAA buoy station GISL1 were used to weight calculations by the cumulative speed of wind gusts for each direction, and satellite imagery was used to approximate *α_j_* for each plot (Bernik, Eppinga, et al., 2018).

We also measured soil attributes and other Earth surface processes related to erosion. In January 2014, soil shear strength was measured to the nearest k Pa using a soil shear vane following Turner (2011). Measurements were taken at the soil surface and at a depth of 10 cm at three locations within each plot. Vertical surface gain (i.e., accretion) and loss (i.e., subsidence, erosion) were measured using a 1‐m steel surface elevation rod that was inserted into the rear section of each plot in December 2012. A face‐down petri dish and attached sedimentation disk were threaded over each rod and fastened flush against the marsh surface. Vertical surface gain and loss were measured to the nearest mm using the distance between the marsh surface and the petri dish. Measurements were taken 3 and 9 months following installation. Accreted material was collected from each sedimentation disk and brought to Tulane University where it was weighed after being dried to a constant mass.

### 2.5 Statistical analyses

We tested for differences in phenotypic traits as well as plant tissue and soil C and N content by conducting nested ANOVAs. To account for hierarchical variance in plant trait measurements, we tested for differences among cores nested within plot and among plots nested within provenance, as well as among provenances. For biomass and C and N content measurements, we conducted analyses of variance among plots nested within provenance and among provenances. Prior to analyses, all variables except for biomass measurements were scaled to standard *z‐*scores with a mean of 0 and standard deviation of 1. For biomass measurements, data were log‐transformed for normality. For variables that were significantly different at the provenance level, we conducted post hoc Tukey HSD pairwise comparison tests to determine the nature of differences according to provenance. This allowed us to evaluate univariate pairwise differences relative to a linear discriminant analysis (LDA) of multivariate phenotypic differentiation conducted by Lumibao et al. (2020).

We also tested for differences in Earth surface processes and soil attributes (Bernik, Eppinga, et al., 2018; Bernik, Pardue, et al., 2018; Silliman et al., 2012). We first described the range of estimated annual erosion rates, which were calculated as the change in plot length, averaged across transects, between September 2012 and 2013 (Silliman et al., 2012; Zengel et al., 2015). We then compared erosion in control and planted plots to assess the influence of planting by conducting an independent samples Mann–Whitney *U* test, which accounts for heterogeneous variance among groups. We also conducted a nested ANOVA to assess variation in angle‐adjusted erosion rate among plots nested within provenance and among provenances, followed by post hoc Tukey tests to assess the nature of differences according to provenance. To assess whether surface elevation differed according to provenance, we conducted separate ANOVAs for data collected in March, September, and November 2013 due to differences in data availability (i.e., because some plots had eroded past the respective elevation pin). A factorial ANOVA was used to assess whether soil shear strength differed according to position (shoreline, interior, between plants), depth (0 cm, 10 cm), and provenance (Bernik, Pardue, et al., 2018), with Tukey tests used for post hoc pairwise comparisons.

We conducted partial least‐squares regression (PLSR) analyses to assess whether angle‐adjusted shoreline erosion rate and soil shear strength reflected variation in individual plant traits. The PLSR analyses were conducted using the pls package (Mevik & Wehrens, 2007) by first performing the analyses with cross‐validation to determine the optimum number of components and then rerunning the model with the ascribed number of components (i.e., linear combinations of the original predictor variables). We report estimated regression coefficients (*r*), with tests for significance based on the jackknife method (Mevik & Wehrens, 2007). Because the CP cultivar was represented by only two plots, we assessed whether its inclusion substantively altered the results of plot‐level analyses. Comparisons of analyses (i.e., with and without the CP plot data) showed that inclusion did not alter our findings—it only resulted in a slight decrease in statistical power. We thus retained the CP plot data in all analyses. Unless otherwise noted, all statistical analyses were carried out in R (R Core Team, 2016).

## 3 RESULTS

### 3.1 Genotypic variation

**FIGURE 2:**
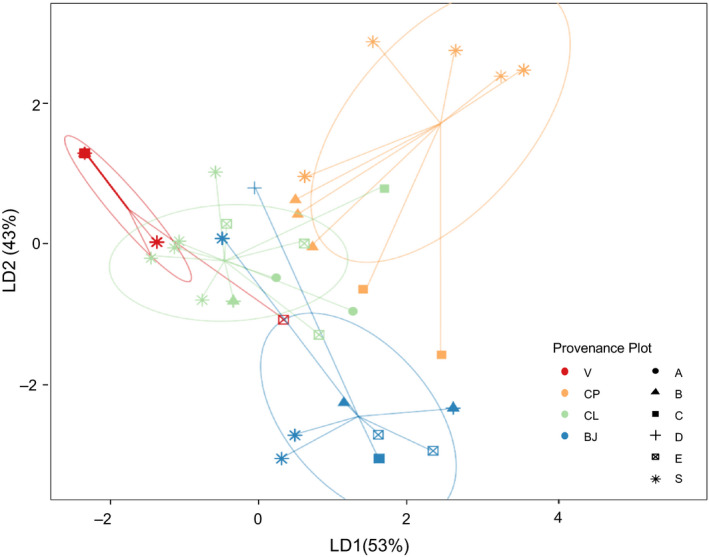
Discriminant analysis of principal components (DAPC) scatterplot of the first two principal components describing allelic variation among *Spartina alterniflora* according to sample location and provenance. Each point represents an individual specimen colored‐coded according to provenance (motif color) and sample location (motif shape; A–E = study plots as per Figure 1; S = source location)

Genotypic variation in the BJ and CL plots was comparable whereas contrasting levels were found in plots planted with the V and CP cultivars (Table S1, Figure 2). We detected a single genotype in all but one sample from V plots, though two additional genotypes were detected in source population samples (Table S1, Figure 2). While the NSU nursery stock is thought to constitute a clonal monoculture, and thus, V plants are expected to exhibit a single genotype (Fine & Thomassie, 2000; LAPMC, 1989), additional genotypic richness might have arisen due to unintentional outcrossing among genetically distinct nursery stocks. In contrast, all but two of the plants sampled from CP plots exhibited distinct genotypes, which is consistent with the origination of the CP line from crosses between several genetically distinct accessions of *S. alterniflora* (Knott et al., 2012, 2013). The BJ and CL plots exhibited comparable genotypic variation among plots, though CL plants exhibited greater overall variation (Table S1, Figure 2). Nearly all of the samples from the BJ and CL plots exhibited a distinct genotype (Table S1, Figure 2). All genotypes were correctly assigned to their corresponding source population according to likelihood values. Global *F*_ST_ was estimated at 0.28 (*p* < .001), with significant pairwise *F*_ST_ values (all, *p* < .0001) among provenances ranging from 0.15 to 0.52. Consistent with this, the DAPC revealed that there is little overlap among genotypes according to provenance (Figure 2).

### 3.2 Phenotypic variation

**FIGURE 3:**
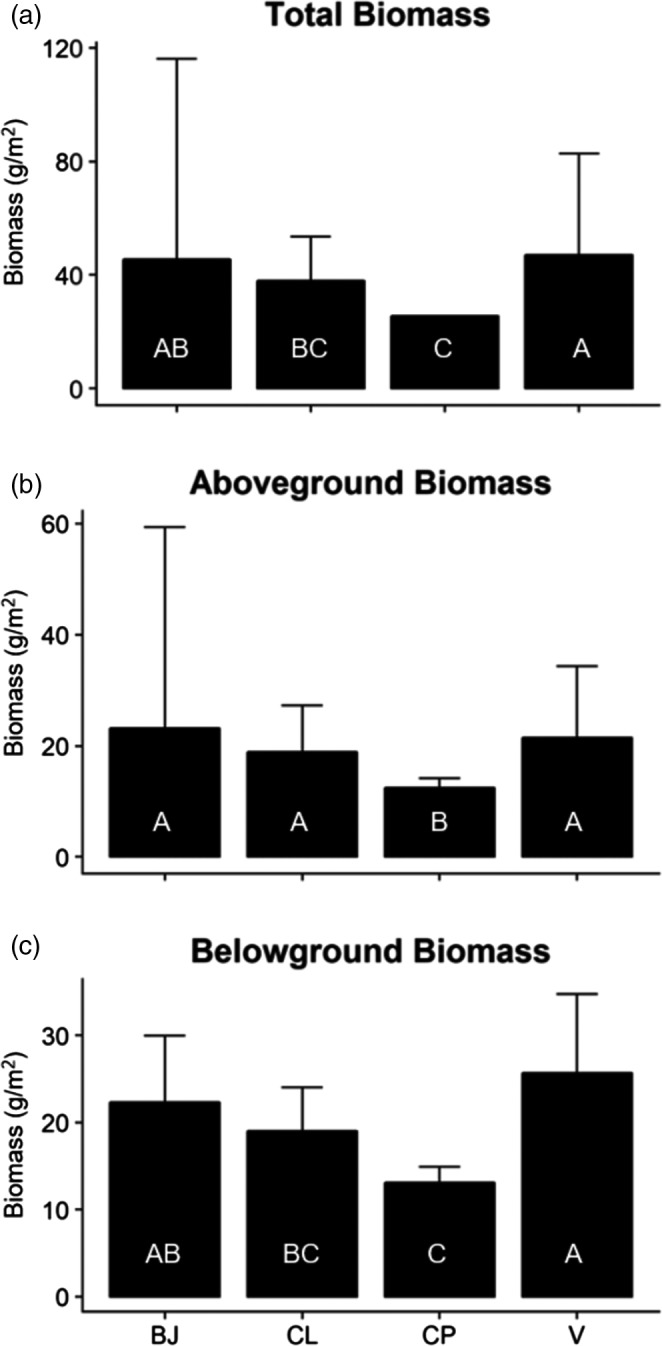
Variation in biomass (mean ± *SE*) exhibited by *Spartina alterniflora* from Bay Jimmy (BJ) and Catfish Lake (CL), as well as by Vermilion (V) and CP (CP) cultivars grown under common garden conditions at the study site. See Table S1 in the Supporting Information for further details on biomass measurements. Different letters inside the bars indicate significant pairwise differences according to provenance

Biomass varied according to provenance (Tables S1 and S2, Figure 3). Post hoc tests illustrated that BJ and V plants produced comparable amounts of total biomass, whereas both produced more total biomass than did CP plants, and V plants produced more total biomass than did CL plants (Figure 3). Considered separately, AG and BG biomass differed according to provenance (*F*_3_ = 6.47, *p* < .001 and *F*_3_ = 5.03, *p* = .010, respectively). AG biomass also differed among plots nested by provenance (*F*_13_ = 4.16, *p* < .001), and post hoc tests revealed that CP plants produced less AG biomass than plants from all other sources (Figure 3). Differences in BG biomass followed the same pattern as total biomass (Table S1, Figure 3), reflecting variation across the 0–10 cm horizon (*F*_3_ = 6.709, *p* = .001) rather than the 10–20 cm horizon (*F*_3_ = 2.225, *p* = .105).

**FIGURE 4:**
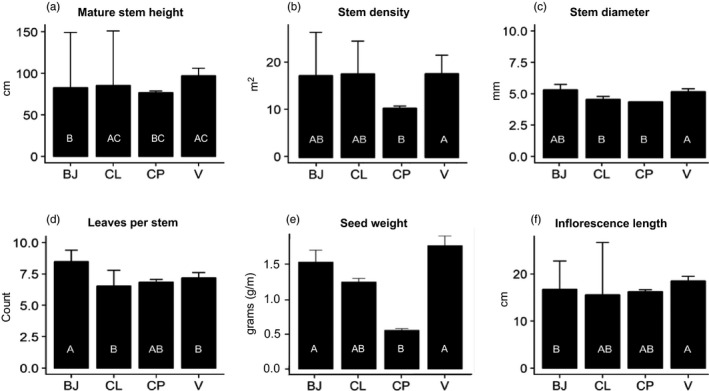
Trait variation (mean ± *SE*) among *Spartina alterniflora* from Bay Jimmy (BJ) and Catfish Lake (CL), as well as in Vermilion (V) and CP (CP) cultivars grown under common garden conditions at the study site. See Table S3 in the Supporting Information for further details on trait measurements. Different letters inside the bars indicate significant pairwise differences according to provenance

Canopy architecture varied according to provenance as well as among plots and cores (Tables S3 and S4, Figure 4). For example, we detected differences in mature stem length by provenance (*F*_3_ = 4.92, *p* < .001) and plot nested by provenance (*F*_13_ = 3.09, *p* < .001). Post hoc tests illustrated that V plants exhibited taller mature stems than did CP plants and BJ plants (Tables S3, Figure 4). Stem diameter varied among provenances (*F*_3_ = 6.24, *p* < .001), plot nested by provenance (*F*_13_ = 4.64, *p* < .001), and core nested by plot (*F*_17_ = 3.44, *p* < .001) (Table S4). Post hoc tests revealed that V plants had thicker shoots than CP and CL plants (Table S3, Figure 4). Both number of leaves per stem and the length of leaves varied according to provenance (*F*_3_ = 5.18, *p* < .001 and *F*_3_ = 4.14, *p* = .01, respectively); BJ plants exhibited more leaves per stem than did V and CL plants (Table S3, Figure 4), whereas BJ and V plants exhibited longer leaves compared to CL plants (Table S4). We also detected variation among plots nested within provenance for leaf number and length (*F*_13_ = 6.40, *p* < .001 and *F*_13_ = 2.09, *p* = .03, respectively, Table S4). No differences were observed in root to shoot (R:S) ratios (Table S4).

We observed significant variation in reproductive traits (Table S4). Total seed weight varied by provenance (*F*_3_ = 4.65, *p* = .01) and among plots nested within provenance (*F*_15_ = 9.86, *p* < .001) with post hoc tests showing that V and BJ plants exhibited significantly larger seed heads than did CP plants (*p* = .006 and *p* = .03, respectively; Table S4, Figure 4). Inflorescence length differed according to provenance (*F*_3_ = 3.85, *p* = .03) and plot nested within provenance (*F*_13_ = 8.33, *p* < .001), with V plants exhibiting longer inflorescences than BJ plants (Table S4, Figure 4).

### 3.3 Tissue and soil composition

**FIGURE 5:**
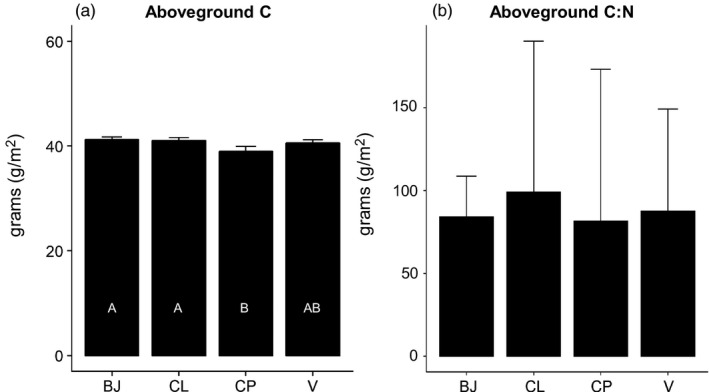
Variation (mean ± *SE*) in carbon (C) and nitrogen (N) in aboveground biomass of *Spartina alterniflora* from Bay Jimmy (BJ) and Catfish Lake (CL), as well as in Vermilion (V) and CP (CP) cultivars grown under common garden conditions at the study site (as per Figure 1). See Table S1 in the Supporting Information for further details on C and N measurements. Different letters inside the bars indicate significant pairwise differences according to provenance

Greater differences were found in tissue than in soil composition according to plant provenance (Table S2, Figure 5). AG tissue C (C_AG_) differed by provenance (*F*_3_ = 5.19, *p* < .001) and by plot nested within provenance (*F*_13_ = 2.78, *p* = .01), with CL and BJ plants exhibiting more than CP (Table S1; Figure 5). Though AG tissue N (N_AG_) was not significantly different (*F*_3_ = 2.61, *p* = .07), CL plants exhibited noticeably lower N_AG_ than other plants, which translated to significant differences in (C:N)_AG_ (*F*_3_ = 3.17, *p* = .04) according to provenance (Table S2, Figure 5), largely reflecting a difference between CL and V plants (Table S1, Figure 5). Neither C_BG_ nor N_BG_ differed by provenance or plot nested within provenance (Table S2). Neither soil C nor soil N difference by provenance (*F*_3_ = 0.48, *p* = .49 and *F*_3_ = 0.02, *p* = .96; Table S1) but both varied by plot (*F*_15_ = 2.75, *p* < .001 and *F*_15_ = 2.26, *p* = .03).

### 3.4 Erosion, soil strength, and marsh elevation

Overall, the marsh shoreline eroded at an average rate of 1.09 m/year. When excluding control plots, the average erosion rate was 0.96 m/year. As expected, erosion progressively declined from west to east (*R* ^2^ = 0.24, *p* = .02), reflecting the gradual curve of the shoreline away from direct exposure to wind‐driven waves (Figure 1). Adjusting for shoreline angle reduced the west–east trend in erosion rates (*R* ^2^ = 0.14, *p* = .08). The rate of angle‐adjusted erosion averaged 2.03 m/year across all plots and 1.74 m/year excluding control plots.

**FIGURE 6:**
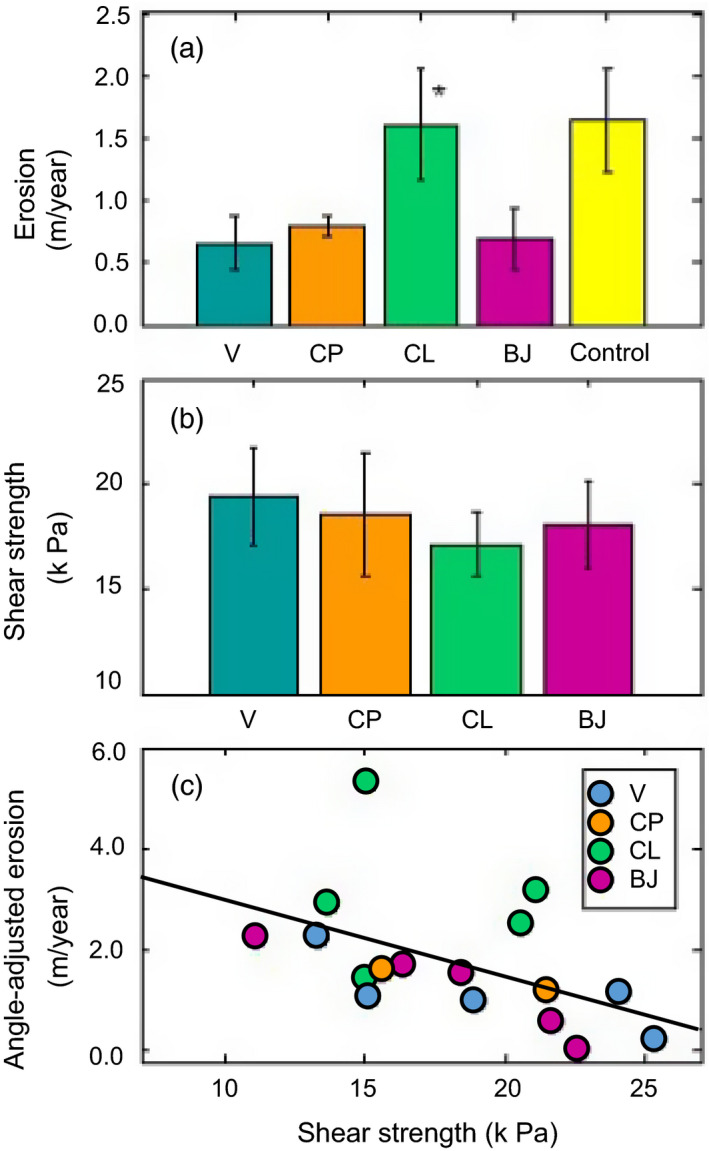
Soil shear strength and erosion for marsh restored with plants from different provenances: Vermilion (V) or CP cultivars, and natural stock from nearby (CL) or local (BJ) populations. (a) Mean erosion across planted and control plots: ‘*’ = indicative of statistical difference between CL and all other planted plots. (b) Mean soil shear strength across planted plots. (c) Significant relationship between soil shear strength and erosion rate with planted plots with reference to provenance

Differences in angle‐adjusted erosion rates reflected plant provenance (*F*_4_ = 16.42, *p* < .001; Figure 6) and plot nested within provenance (*F*_17_ = 8.94, *p* < .001). Overall, plots with CL plants eroded more quickly than all other planted plots; CL plots eroded an additional 1.88 m/year on average (Figure 6). Control plots eroded an additional 1.30 m/year on average than planted plots (*F*_1_ = 3.13, *p* = .09), and an additional 1.85 m/year on average if CL plots was excluded from consideration (*F*_1_ = 8.60, *p* = .01).

Shear strength differed by depth (*F*_1_ = 51.59, *p* < .001, partial *η* ^2^ = 0.41) and location (*F*_2_ = 5.86, *p* = .004, partial *η* ^2^ = 0.14). Significantly higher values were obtained at the surface compared to values at 10 cm depths. Shear strength also was higher within the footprint of plants in plot interiors than at the shoreline edge or areas between plants. While there was no effect of plant provenance (*F*_3_ = 0.22, *p* = .88), variation in soil shear strength tracked differences in erosion rate (Figure 6), and shear strength explained a significant amount of the variation observed in erosion rate (*F*_1_ = 4.85, *R* ^2^ = 0.24, *p* = .04; Figure 6).

All plots experienced vertical surface loss, but no differences were found according to plant provenance (Mar. 2013: *F*_3_ = 0.73, *p* = .55; Sep. 2013: *F*_3_ = 1.28, *p* = .35; Nov. 2013: *F*_3_ = 2.27, *p* = .14). We did, however, observe an emerging trend that might have resulted in significant differences had monitoring continued over a longer timeframe.

PLSR analyses recovered significant associations between plant traits and both angle‐adjusted erosion rate and soil shear strength. PLSR analyses revealed that plant traits aggregately explain 59.9% of variation in angle‐adjusted erosion rates and 66.5% of shear strength variation. PLSR analyses identified BG biomass (*r* = −.20, *p* = .025), total biomass (*r* = −.14, *p* = .017), tiller density (*r* = −.12, *p* = .036), tiller height (*r* = .23, *p* = .005), and (C:N)_AG_ (*r* = .24, *p* = .029) as predictors of angle‐adjusted erosion rate. PLSR analysis identified mature stem height (*r* = .13, *p* = .002), tiller height (*r* = .07, *p* = .015), stem diameter (*r* = .1400132, *p* = .002), leaf number (*r* = .1485124, *p* < .001), and inflorescence length (*r* = .1302838, *p* < .001) as predictors of soil shear strength.

## 4 DISCUSSION

The possibility of ecosystem consequences arising from intraspecific variation is seldom considered in management applications of engineering species (Blum et al., 2014; Strong & Ayres, 2013). To explore the implications of this concern, we examined how erosion and related soil properties varied across a salt marsh shoreline planted with *S. alterniflora* from four genetically distinct source populations (Figure 2) exhibiting heritable differences in traits that can mediate the stability and transport of Earth surface materials (Bernik, Eppinga, et al., 2018; Bernik, Pardue, et al., 2018; Lumibao et al., 2020). Consistent with prior work showing that salt marsh ecosystem attributes can differ according to provenance (Bernik, Pardue, et al., 2018; Seliskar et al., 2002), we found that shoreline erosion rates differed according to intraspecific variation in *S. alterniflora* (Figure 6). Observed variation in shoreline erosion rates reflected variation in functional traits (e.g., stem height, stem density) (Tables S3 and S4, Figure 4) that can dampen wind‐wave energy (Lei & Nepf, 2019) as well as variation in other attributes like biomass and nutrient uptake that can influence soil integrity (Tables S1 and S2, Figures 3 and 5). These findings illustrate that use of *S. alterniflora* from different source populations can result in different restoration outcomes. Evidence that the effect of provenance was, in some instances, equivalent or larger than the effect of planting further highlights the importance of considering intraspecific variation when using landform engineers to create, shape, or improve habitat.

### 4.1 Genotypic and heritable trait variation

Evidence of morphological trait and tissue nutrient differences among cultivated, local, and nonlocal populations under common garden conditions corroborates prior findings of heritable phenotypic variation in *S. alterniflora*. Affirming our prior work (Bernik, Eppinga, et al., 2018), we found that plants from the different sources exhibited distinct combinations of trait differences (Tables S1–S4, Figures 3, 4, 5). Pairwise comparisons elaborate overall patterns of multivariate phenotypic differentiation in canopy architecture, biomass production, and tissue recalcitrance (Lumibao et al., 2020). For example, differences in a suite of stem traits affirm that BJ and V plants exhibit particularly distinct canopy architectures (Lumibao et al., 2020). A field‐scale common garden experiment comparing *S. alterniflora* from different regions of the Atlantic coast similarly found evidence of heritable variation in biomass production, canopy architecture, as well as belowground trait variation (Seliskar et al., 2002). Likewise, heritable differences in biomass and height were found among genotypes from proximate sites within a southwestern Louisiana embayment (Proffitt et al., 2005; Travis & Grace, 2010). Qing et al. (2012) also detected heritable differences in N uptake and allocation between *S. alterniflora* introduced to China as well as among genotypes from native source populations, illustrating that functional traits and resource use may rapidly evolve in response to local selective pressures.

Comparison of our findings to those of a sister greenhouse experiment (Bernik, Pardue, et al., 2018) highlights the importance of assessing plant performance under field‐scale conditions. Our greenhouse‐scale common garden study (Bernik, Pardue, et al., 2018) likewise detected heritable variation in biomass production, canopy architecture, and nutrient use among plants from the same populations used in this study. Notably, both studies detected parallel differences in trait variation according to provenance. For example, both studies found that CL plants exhibit longer shoots (exclusive of tillers; Table S3) and fewer leaves per shoot than do BJ plants (Table S3, Figure 4). Some measures of plant performance differed, however, depending on whether plants were grown in the field or greenhouse. For instance, field‐grown CL plants had lower N whereas V and CP plants had more N in AG tissues compared to plants grown under greenhouse conditions. Additionally, BJ plants produced relatively more biomass while CL and CP plants produced less biomass when grown under field‐scale conditions. Though this discrepancy may in part be due to phenotypic plasticity (Bernik, Pardue, et al., 2018), it also suggests that BJ plants are better adapted to local conditions compared to nonlocal plants and cultivars. In addition, it supports findings from other studies (Proffitt et al., 2005; Travis & Grace, 2010) suggesting that local adaptation in *S. alterniflora* can vary within and among nearby coastal embayments that harbor genetically distinct populations (Blum et al., 2007; Hughes & Lotterhos, 2014; Novy et al., 2010; Richards et al., 2004; Utomo et al., 2009).

Contrary to expectation, we found that cultivars did not always outperform or surpass local and nonlocal plants at characteristic attributes. The V cultivar was expected to exhibit greater biomass productivity, for example, but we found that plants from the local natural population produced comparable biomass. The CP cultivar was expected to exhibit greater fecundity, yet we found that it tended to produce fewer seed heads and that it exhibited lower average seed weight compared to plants from the other populations included in this study. Notably, these findings depart from those of our prior greenhouse study. When evaluating the same set of source populations, Bernik, Pardue, et al. (2018) found that the V cultivar consistently produced the highest average biomass, and though the V cultivar also produced the largest number of seed heads, the CP cultivar produced the greatest number of seeds per seed head. Despite there being notable parallels between field and greenhouse measures of trait variation (including measures indicating that the CP cultivar exhibits trade‐offs between growth and reproduction), these inconsistencies suggest that deviations from expectations may arise due to selective or plastic responses, or some combination thereof, to local environmental conditions. This raises the possibility that unanticipated trait expression or performance might consequently translate to unexpected ecosystem outcomes, including marsh platform instability (Bernik, Eppinga, et al., 2018). So long as adequate precautions are taken to prevent unintentional outcrossing, it would be prudent for cultivation and management programs seeking to improve targeted traits and ecosystem attributes (respectively) to perform in situ trials at sites slated for restoration in order to evaluate the suitability of donor sources and to determine whether performance targets can be achieved under local environmental conditions. Evidence that the V cultivar exhibits greater than expected genotypic richness (i.e., more than a single genotype) also highlights the potential value of screening nursery stocks to assess the possibility of outcrossing, which might lead to unanticipated outcomes of restoration projects due to uncharacteristic functional trait variation (Bernik et al., 2016).

### 4.2 The extended phenotype of a landform engineer

Our findings illustrate that Earth surface processes and attributes are sensitive to intraspecific variation in a well‐recognized, widely distributed, and widely used landform engineer. Our study offers further evidence that the stability and transport of Earth surface materials can be mediated by plants (Corenblit et al., 2011) and that plants exhibit intraspecific variation in their capacity to reduce surface erosion (Berendse et al., 2015; Bernik, Eppinga, et al., 2018; Bernik, Pardue, et al., 2018). Our results are consistent with prior work showing that soil shear strength varies according to plant provenance (Bernik, Pardue, et al., 2018). We also found that shoreline erosion rates reflect provenance, which supports the idea that biogeomorphic feedbacks can be mediated by adaptive trait variation in plants (Bernik, Pardue, et al., 2018; Corenblit et al., 2011).

The observed differences in erosion rates (Figure 6) and trends in soil shear strength indicate that intraspecific variation in *S. alterniflora* influences marsh shoreline stability. Consistent with the results of our prior greenhouse study (Bernik, Pardue, et al., 2018) as well as associated theory and simulations (Bernik, Eppinga, et al., 2018), we found that *S. alterniflora* exhibits heritable variation in traits that can influence the stability and transport of Earth surface materials. Regression analyses revealed that soil shear strength and erosion are influenced by heritable variation in aspects of canopy architecture (e.g., stem height, stem density, stem diameter) that can dissipate wave energy (Lei & Nepf, 2019; Leonard & Luther, 1995; Nepf, 1999; Paul et al., 2012). Regression analyses also provided support for prior work showing that BG growth mediates soil erodibility and shoreline stability (Bernik, Pardue, et al., 2018; Deegan et al., 2012; Gyssels et al., 2005; Howes et al., 2010; Turner et al., 2002; van Eerdt, 1985). We detected a significant negative relationship between erosion and BG biomass, indicating that greater BG biomass production reduces erosion, and thus promotes greater marsh resilience to wind‐driven wave action (Turner et al., 2002). It is important to note, however, that some of our other findings suggest erosion rates do not necessarily correspond to coarse measures of BG production. For example, we found that plots with plants producing lower average BG biomass did not always experience greater erosion (Table S1; Figures 3 and 6). This ostensibly contrary observation is consistent with findings from our prior greenhouse‐scale experiment (Bernik, Pardue, et al., 2018) indicating that heritable variation in BG architecture, like the ratio of fine root to rhizome production, mediates the influence of BG biomass on soil erodibility (Bernik, Pardue, et al., 2018). Thus, soil erodibility could be lower when plants produce less BG biomass if, for example, individual roots become less impactful as root diameter increases (i.e., where smaller roots contribute greater strength per unit area (Baets et al., 2007; van Eerdt, 1985).

Some of our findings suggest that shoreline erosion may also be attributable to physiological differences in resource allocation. A trend toward lower C:N ratios in BG tissue of *S. alterniflora* in plots that experienced higher erosion (particularly compared to plants with lower BG biomass in plots exhibiting lower erosion) provides some support for this hypothesis (Table S3; Figures 3 and 5). If the relative strength of narrower roots reflects a smaller volume of area dominated by tissue composed of structural carbohydrates (Genet et al., 2005; Striker et al., 2007), then lower C:N ratios could indicate the presence of thicker but less dense root networks, where overall root tensile strength and soil shear strength are comparably low (Bernik, Pardue, et al., 2018).

It is also possible that erosion rates reflect differences in physiological responses to stress. Stress responses can alter soil shear strength by modifying plant architecture, including root density and root size. Stress responses also can alter soil chemistry by reducing lignin content of litter or increased oxygenation, which can reduce soil shear strength by increasing rates of decomposition (Morris et al., 2013). Though C:N ratios varied, plants from all source populations exhibited relatively high ratios in AG and BG tissue, with values of N concentrations among the lowest reported in the literature (McIntire & Dunstan, 1976). This suggests that all the plants examined in this experiment were nutrient limited, perhaps because resource uptake was impaired by residual hydrocarbon contamination or salinity stress (Bradley & Morris, 1990; Osgood & Zieman, 1993; Wright et al., 1996). If so, then the relatively lower N concentration and higher C:N ratios exhibited in AG tissue by CL plants—which corresponded to plots with the highest erosion rate—would suggest that CL plants were more nutrient limited and less capable of responding to environmental stressors than plants from the other source populations. Though further work focusing on bioavailable forms of N is warranted, some traits exhibited by CL plants (e.g., comparably low root to shoot ratios) indicate that responses may be due to lower nutrient transport efficiency, which would result in reduced N uptake, reduced transport of N to AG tissue, and relatively higher N concentrations in roots (Table S1). Notably, indications of stress response do not appear to be related to residual hydrocarbon contamination at the study site. Post hoc analyses found no relation between AG or BG tissue N concentrations and residual total PAH concentrations in the soil (Pearson's *r* = .23, *p* = .19; *r* = −.19, *p* = .31; data available upon request; Lumibao et al., 2018, 2020), which did not differ among the study plots according to provenance (ANOVA, *F*_3_ = 2.49, *p* = .06). Residual soil PAH concentrations also were not associated with either erosion or soil shear strength (Pearson's *r* = −.09, *p* = .55; *r* = .16, *p* = .34). Nonetheless, further assays of plant and soil chemistry (e.g., bioavailable forms of N, porewater conditions) could help resolve the nature of intraspecific variation observed in tissue nutrient content associated with resource uptake in *S. alterniflora* (Elsey‐Quirk et al., 2011) and thereby better elucidate architectural characteristics and geochemical conditions influencing erosion.

### 4.3 Implications for environmental restoration

Our findings underscore concerns that the use of landform engineers from nonlocal sources, including cultivated nursery stocks, for coastal restoration can potentially lead to unanticipated and undesirable outcomes (Bernik et al., 2016; Strong & Ayres, 2013). Unexpected results might arise because the performance of plants introduced for restoration departs from expectation or because selective advantages might be sufficient for introduced plants to overwhelm surrounding natural populations (Strong & Ayres, 2013). While the performance of one cultivar (V) examined in this study adhered to most expectations, the use of nonlocal source materials for coastal restoration nonetheless warrants careful consideration, including determining best practices that can be adopted to reduce or avoid complications.

Our results indicate that use of *S. alterniflora* cultivars can modulate outcomes of marsh restoration, which notably contrasts with findings from studies of prairie grassland restoration. As in marsh restoration, cultivars are often used for prairie restoration. Comparisons of plants from wild and cultivated seeds for a range of prairie grasses have not detected differences in ecosystem attributes like net primary productivity, C accrual, and N mineralization (Baer et al., 2014; Gibson et al., 2013; Wilsey, 2010). It is possible that inconsistencies between the use of cultivars for restoration of prairies and salt marshes are a consequence of prairies harboring greater species diversity, which can reduce the relative influence of any one species on ecosystem attributes (Baer et al., 2014). It is also possible that prairie cultivars better reflect natural populations (e.g., by harboring more representative genetic variation) than do salt marsh cultivars (e.g., V cultivars; Figure 2).

Further evidence that plant performance is subject to prevailing environmental conditions (Bernik, Eppinga, et al., 2018; Bernik, Pardue, et al., 2018) affirms the importance of understanding intraspecific variation in landform engineering. This is especially important for restoration projects that will alter stressor exposure or resource availability, like the massive river diversions that are expected to deliver sediment and nutrient‐laden fresh water to the coastal bays of the lower Mississippi River Delta. It remains unclear whether increased nutrient delivery to coastal marshes will help reduce erosion (i.e., by stimulating BG productivity; Morris et al., 2013) or accelerate erosion (i.e., by suppressing BG biomass and increasing decomposition; Turner, 2011). Our findings indicate that erosion rates may shift under different nutrient regimes because of plastic and heritable variation in resource acquisition and use. Likewise, our greenhouse‐scale common garden study (Bernik, Eppinga, et al., 2018) demonstrated that *S. alterniflora* exhibits plastic and heritable responses to elevated nutrients, including greater biomass production and shifts in BG attributes (e.g., root to rhizome ratios, rhizome tensile strength), that result in higher soil erosion resistance. Accordingly, circumscribing intraspecific variation in landform engineers at candidate outfall locations could improve predictive models intended to characterize ecosystem outcomes of river diversions. Models also could be improved by accounting for a larger portfolio of traits that can modify soil and shoreline characteristics (Bernik, Eppinga, et al., 2018; Bernik, Pardue, et al., 2018).

Evidence that nonlocal plants exhibit reduced capacity to buffer against erosion suggests that modifying the genetic composition of *S. alterniflora* populations could give rise to eco‐evolutionary feedbacks that iteratively influence the form and fate of marsh ecosystems. Work on other foundational plants, like *Populus* trees, illustrates that ecosystems can be shaped by eco‐evolutionary feedbacks. For instance, selection pressures from an external driver (e.g., beavers) can shift the genetic composition of *Populus* stands, altering rates of N mineralization and decomposition, which can further influence stand composition (Schweitzer et al., 2008). Positive eco‐evolutionary feedbacks also have been implicated in the aggressive spread of *Phalaris arundinacea*, whereby genotypes that require less N produce a more recalcitrant litter layer that can tie up N supply, a condition favorable to said genotypes (Eppinga et al., 2011). Our findings suggest that the introduction of nonlocal plants for restoration might elevate shoreline erosion, which could result in a negative feedback if a combination of outcrossing and increased land loss eliminates alleles for genetically based traits that reduce erosion from the local gene pool. Introducing cultivars might similarly give rise to feedbacks that alter ecosystem trajectories. Negative feedbacks might arise if cultivation results in fitness trade‐offs (e.g., growth vs. reproduction) or if it inadvertently influences the expression of nontarget traits. Subsequent alteration of the local gene pool (e.g., via outcrossing) might result in declining function and possibly even ecosystem failure (e.g., via elevated erosion rates). Steps can be taken, however, to prevent or disrupt unfavorable feedback cycles. For example, genomic monitoring of plants in restored and surrounding marshes could offer guidance to structure interventions sufficient to correct the course of ecosystem evolution toward stability and persistence.

## CONFLICT OF INTEREST

None declared.

## Supporting information

Supplementary Material

## Data Availability

Data are publicly available through the Dryad Digital Repository and the Gulf of Mexico Research Initiative Information & Data Cooperative (GRIIDC).
