# Supplementary material for: Intraspecific variation in landform engineering across a restored salt marsh shoreline
Source: Evol Appl. 2020 Nov 7;14(3):685–97. doi: 10.1111/eva.13148 (PMC7980261; doi:10.1111/eva.13148)
Supplement: Supplementary file 1 — Supplementary Material [file EVA-14-685-s001.docx]

*Evolutionary Applications*

Supporting Information for

**Intraspecific variation in landform engineering across a restored salt marsh shoreline**

**TABLES**

**Table S1.** Genetic variation, plant productivity, plant tissue composition, and soil composition for a marsh restored with plants of different provenance: cultivars developed for biomass (V) and seed-production (CP), and natural populations from nearby (CL) and local to the study site (BJ). Measures include mean genotypic richness per plot (*G*_P_) and overall (*G*), aboveground (AG) and belowground (BG) biomass in grams (g), including BG components by depth (in centimeters). Carbon (C) and nitrogen (N) measures are given as percent dry weight. Sample numbers (*N*) for different measures are distinguished by subscripts. Standard deviations are given in parentheses.

|  | Genetics Biomass (g) | | | | | | BG Biomass (g) | | |  | | AG tissue (%) | | | | | BG tissue (%) | | | | | Soil (%) | | | |
| --- | --- | --- | --- | --- | --- | --- | --- | --- | --- | --- | --- | --- | --- | --- | --- | --- | --- | --- | --- | --- | --- | --- | --- | --- | --- |
|  | *N* | G_P_ | G | AG | BG | Total | 0-10 | 10-20 | R:S | | *N_A_* | | C | N | C:N | *N_B_* | | C | N | C:N | *N_S_* | | C | N |  |
| V | 31 | 1.2 | 4 | 21.5 | 27 | 48.4 | 18.6 | 8.4 | 1.3 | | 15 | | 40.2 | 0.5 | 84.3 | 15 | | 34.9 | 0.4 | 109.4 | 15 | | 13.2 | 0.6 |  |
|  |  | (0.4) |  | (7.6) | (9.8) | (14.3) | (5.4) | (5.0) | (0.5) | |  | | (1.2) | (0.1) | (17.4) |  | | (7.9) | (0.2) | (57.8) |  | | (5.8) | (0.1) |  |
| CP | 22 | 2.5 | 10 | 12.4 | 13.1 | 25.5 | 7.9 | 4.3 | 1.2 | | 6 | | 39 | 0.5 | 86.9 | 6 | | 40.1 | 0.4 | 124.9 | 6 | | 12.8 | 0.5 |  |
|  |  | (0.7) |  | (5.3) | (2.6) | (5.4) | (2.3) | (2.5) | (0.5) | |  | | (3.2) | (0.1) | (33.1) |  | | (1.9) | (0.1) | (38.1) |  | | (6.5) | (0.2) |  |
| CL | 31 | 1.6 | 12 | 20.3 | 17.4 | 36.3 | 12.3 | 5.5 | 1.0 | | 15 | | 41.3 | 0.4 | 104.8 | 10 | | 36.8 | 0.5 | 93.5 | 12 | | 11.4 | 0.5 |  |
|  |  | (0.9) |  | (6.4) | (6.6) | (11.0) | (5.2) | (2.7) | (0.5) | |  | | (1.3) | (0.1) | (23.6) |  | | (5.6) | (0.2) | (34.9) |  | | (5.8) | (0.1) |  |
| BJ | 21 | 1.8 | 9 | 23.1 | 21.8 | 45.0 | 15.5 | 6.3 | 1.0 | | 15 | | 41.1 | 0.5 | 86.9 | 14 | | 37.7 | 0.4 | 103.7 | 15 | | 12.4 | 0.6 |  |
|  |  | (0.8) |  | (10.2) | (7.6) | (16.0) | (6.0) | (2.7) | (0.5) | |  | | (1.4) | (0.1) | (21.9) |  | | (6.1) | (0.2) | (38.1) |  | | (3.8) | (0.1) |  |

**Table S2.** Nested ANOVA analyses of variation in biomass (top panel) and tissue composition (bottom panel) according to provenance and plot (nested within provenance). Bold P values indicates P < 0.05. Abbreviations and values of traits for each provenance are provided in Table S1.

|  | **df** | **SumSq** | **MeanSquare** | **F-value** | **P** |
| --- | --- | --- | --- | --- | --- |
| **BIOMASS** | | | | | |
| **TOTAL** |  |  |  |  |  |
| Provenance | 3 | 1.95 | 0.65 | 7.26 | **0.00** |
| Plot | 12 | 2.05 | 0.17 | 1.90 | 0.07 |
| **AG** |  |  |  |  |  |
| Provenance | 3 | 1.69 | 0.56 | 6.47 | **0.00** |
| Plot | 13 | 4.71 | 0.36 | 4.16 | **0.00** |
| **BG** |  |  |  |  |  |
| Provenance | 3 | 1075.10 | 358.40 | 5.03 | **0.01** |
| Plot | 12 | 541.50 | 45.10 | 0.63 | 0.80 |
|  |  |  |  |  |  |
| **TISSUE COMPOSITION** | | | | | |
| **C_AG_** |  |  |  |  |  |
| Provenance | 3 | 0.02 | 0.01 | 5.19 | **0.00** |
| Plot | 13 | 0.04 | 0.00 | 2.78 | **0.01** |
| **N_AG_** |  |  |  |  |  |
| Provenance | 3 | 0.37 | 0.12 | 2.61 | 0.07 |
| Plot | 13 | 0.51 | 0.04 | 0.84 | 0.62 |
| **C_BG_** |  |  |  |  |  |
| Provenance | 3 | 0.14 | 0.05 | 1.27 | 0.30 |
| Plot | 12 | 0.80 | 0.07 | 1.78 | 0.10 |
| **N_BG_** |  |  |  |  |  |
| Provenance | 3 | 1.65 | 0.55 | 0.56 | 0.65 |
| Plot | 12 | 11.24 | 0.94 | 0.96 | 0.51 |
| **C_BG_:C_AG_** |  |  |  |  |  |
| Provenance | 3 | 0.18 | 0.06 | 1.62 | 0.21 |
| Plot | 12 | 0.82 | 0.07 | 1.80 | 0.10 |
| **N_BG_:N_AG_** |  |  |  |  |  |
| Provenance | 3 | 0.718 | 0.2394 | 0.228 | 0.88 |
| Plot | 12 | 11.673 | 0.9728 | 0.927 | 0.53 |
| **(C:N)_AG_** |  |  |  |  |  |
| Provenance | 3 | 0.46 | 0.15 | 3.17 | **0.04** |
| Plot | 13 | 0.75 | 0.06 | 1.18 | 0.33 |
| **(C:N)_BG_** |  |  |  |  |  |
| Provenance | 3 | 1.895 | 0.6315 | 0.632 | 0.60 |
| Plot | 12 | 12.752 | 1.0627 | 1.063 | 0.42 |

**Table S3.** Phenotypic traits for plants from different provenances: cultivars developed for biomass (V) and seed-production (CP), and natural populations from nearby (CL) and local to the study site (BJ). Core measures are averages for all sampled cores, where the number of cores is given as *N*, mature shoots refer to those with seed heads, and total seed weight is denoted *M*. Stem measures are averages for all sampled stems pooled across all cores, where the number of stems sampled is given as *N*_ss_, including: stem diameter (*d*), and average lengths (*L*) of leaves and inflorescences (inflor.).

**Table S4**. Nested ANOVA analyses of variation in shoot traits and seed weight according to provenance, plot (nested within provenance) and core (nested within plot). Bold P values indicates P < 0.05. Abbreviations and values of traits are provided in Table S3.

| **SHOOT** | | | | | |
| --- | --- | --- | --- | --- | --- |
|  | **Df** | **SumSq** | **MeanSquare** | **F-value** | **P** |
| **Stem diameter** |  |  |  |  |  |
| Provenance | 3 | 19.35 | 6.45 | 6.24 | **0.00** |
| Plot | 13 | 62.32 | 4.79 | 4.64 | **0.00** |
| Core | 17 | 60.48 | 0.56 | 3.44 | **0.00** |
| **Leaf number** |  |  |  |  |  |
| Provenance | 3 | 46.60 | 15.54 | 5.18 | **0.00** |
| Plot | 13 | 249.30 | 19.18 | 6.40 | **0.00** |
| Core | 17 | 79.40 | 4.67 | 1.56 | 0.09 |
| **Leaf length** |  |  |  |  |  |
| Provenance | 3 | 545.40 | 181.80 | 4.14 | **0.01** |
| Plot | 13 | 1193.20 | 91.79 | 2.09 | **0.03** |
| Core | 15 | 934.70 | 62.31 | 1.42 | 0.18 |
| **Inflourescence length** |  |  |  |  |  |
| Provenance | 3 | 217.00 | 72.20 | 3.05 | **0.03** |
| Plot | 13 | 2560.00 | 196.96 | 8.33 | **0.00** |
| Core | 17 | 523.00 | 30.78 | 1.30 | 0.20 |
| **Mature stem length** |  |  |  |  |  |
| Provenance | 3 | 7859.00 | 2619.60 | 4.92 | **0.00** |
| Plot | 13 | 21381.00 | 1644.70 | 3.09 | **0.00** |
| Core | 17 | 13943.00 | 820.20 | 1.54 | 0.09 |
| **Stem length 30+ cm** |  |  |  |  |  |
| Provenance | 3 | 7334.00 | 2444.80 | 3.12 | **0.03** |
| Plot | 13 | 27680.00 | 2129.30 | 2.72 | **0.00** |
| Core | 17 | 23964.00 | 1409.60 | 1.80 | **0.03** |
| **Stem length <30cm** |  |  |  |  |  |
| Provenance | 3 | 860.00 | 286.80 | 5.07 | **0.00** |
| Plot | 13 | 1556.00 | 119.70 | 2.11 | **0.01** |
| Core | 17 | 881.00 | 51.80 | 0.92 | 0.56 |
| **Stem density** |  |  |  |  |  |
| Provenance | 3 | 7.21 | 2.40 | 2.82 | **0.05** |
| Plot | 13 | 13.54 | 1.04 | 1.22 | 0.31 |
| **Tiller density** |  |  |  |  |  |
| Provenance | 3 | 4.97 | 1.66 | 1.80 | 0.17 |
| Plot | 13 | 13.71 | 1.05 | 1.15 | 0.36 |
| **Seed weight** |  |  |  |  |  |
| Provenance | 3 | 6.14 | 2.05 | 4.65 | **0.01** |
| Plot | 13 | 56.36 | 4.34 | 9.86 | **0.00** |
| **Root:Shoot ratio (R:S)** |  |  |  |  |  |
| Provenance | 3 | 1.05 | 0.35 | 1.73 | 0.18 |
| Plot | 12 | 3.92 | 0.33 | 1.62 | 0.14 |

**FIGURES**

**Figure S1.**


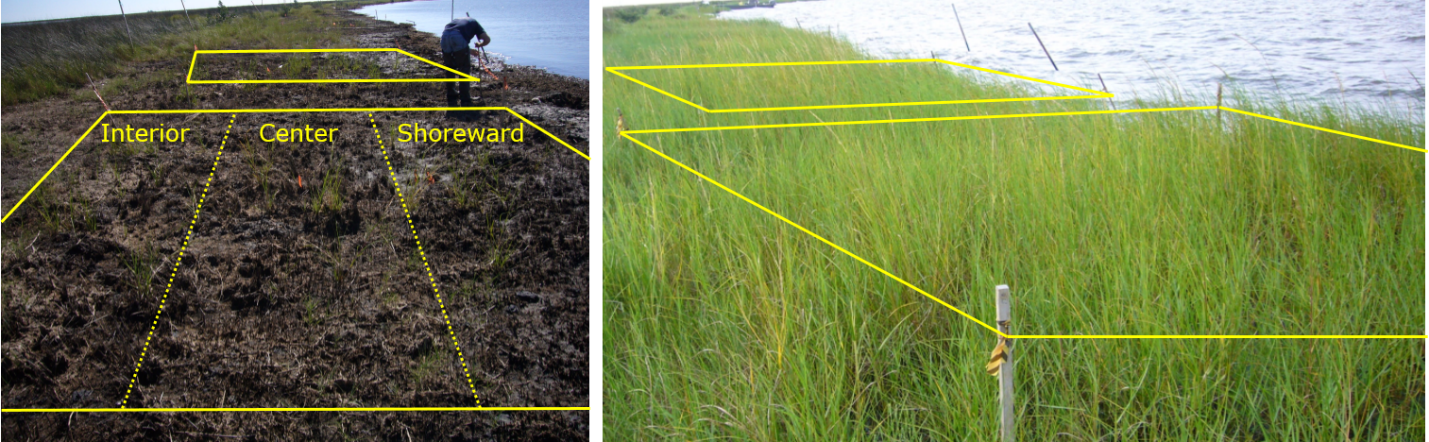


**Figure S1.** Examples of two study plots in November 2011 and August 2012, showing how plant growth progressed from the initial plot layout and planting design. Fifty-five initial starter plants (pictured left) expanded to a dense meadow after one growing season (pictured right).
